# The Evaluation of Restored Proximal Contact Areas with Four Direct Adherent Biomaterials: An In Vitro Study

**DOI:** 10.3390/jfb16040128

**Published:** 2025-04-03

**Authors:** Elena-Cristina Marcov, Mihai Burlibașa, Narcis Marcov, Florentina Căminișteanu, Andreea Angela Ștețiu, Mircea Popescu, Radu-Cătălin Costea, Raluca Mariana Costea, Liliana Burlibașa, Andi Ciprian Drăguș, Maria Antonia Ștețiu, Dana Cristina Bodnar

**Affiliations:** 1 Department of Operative Dentistry, Faculty of Dentistry, “Carol Davila” University of Medicine and Pharmacy, 010221 Bucharest, Romania; elena.marcov@umfcd.ro (E.-C.M.); dana.bodnar@umfcd.ro (D.C.B.); 2 Department of Dental Technology, Faculty of Midwifery and Nursing, “Carol Davila” University of Medicine and Pharmacy, 050474 Bucharest, Romania; mihai.burlibasa@umfcd.ro (M.B.); florentina.caministeanu@drd.umfcd.ro (F.C.); radu-catalin.costea@umfcd.ro (R.-C.C.); andi.dragus@drd.umfcd.ro (A.C.D.); 3 Faculty of Medicine, Lucian Blaga University of Sibiu, 550024 Sibiu, Romania; andreea.stetiu@ulbsibiu.ro (A.A.Ș.); mariaantonia.stetiu@ulbsibiu.ro (M.A.Ș.); 4 Doctoral School, “Carol Davila” University of Medicine and Pharmacy, 37 Dionisie Lupu Street, 020021 Bucharest, Romania; 5 S.C Dentexpert Magic, 050514 Bucharest, Romania; dr.ralucacostea@dentexpert-magic.ro; 6 Genetics Department, Faculty of Biology, University of Bucharest, 060101 Bucharest, Romania; liliana.burlibasa@bio.unibuc.ro

**Keywords:** adherent biomaterials, direct restorations, tightness of proximal contact

## Abstract

The aim of this study was to compare the interproximal contact tightness of lateral teeth after restoring adjacent proximal walls with four types of direct adherent biomaterials. Distal and mesial boxes were prepared on 160 artificial right first and second upper molars. Each set of 40 pairs of boxes was restored using one bulk biomaterial: Equia Forte Fil HT (GC), Cention^®^ Forte (IVOCLAR VIVADENT), Admira Fusion x-tra (VOCO), or 3M^TM^Filtek^TM^ One Bulk Fill. The mean difference in the passing-through force varied from sound to restored surfaces immediately after application, as well as at 7 and 14 days after: Equia Forte Fil HT—4.07 ± 0.01, 4.08 ± 0.01, and 4.11 ± 0.01; Cention^®^ Forte—3.30 ± 0.01, 3.50 ± 0.01, and 3.56 ± 0.01; Admira Fusion x-tra—4.10 ± 0.01, 4.13 ± 0.01, and 4.13 ± 0.01; 3M^TM^Filtek^TM^ One Bulk Fill—4.08 ± 0.01, 4.09 ± 0.01, and 4.07 ± 0.01 (*p* < 0.05). The passing-through force of the restored contact areas showed significantly higher values when compared to those for the sound surfaces, and among them, all biomaterials presented similar values, except for Cention^®^ Forte. The potential clinical relevance of this study relates to better knowing the most appropriate restorative material for large proximal caries on adjacent surfaces from the outset of the treatment protocol.

## 1. Introduction

The integrity and correct direct restoration of the proximal surfaces of teeth and the tightness of the contact between them are very important for the health of the hard dental tissues and the surrounding soft gum area, as well as for proper development of the mastication process. The contact areas disperse the horizontal forces resulting from the decomposition of occlusal pressures, maintaining the continuity of the dental arch and preventing the impact of food on the gingival papilla, leading to potential periodontal complications, the occurrence of secondary caries, and changes in the position of the teeth [1].

Depending on the tooth, the proximal areas have different vertical, sagittal, and transversal configurations, and the contact areas between teeth have different locations.

As a general rule, the mesial contact area is always closer to the occlusal plane than the distal one. Vertically, for the posterior teeth, the contact areas are located at the junction of the occlusal third of the proximal surface with the middle third while, in the transversal direction, they are located at the junction of the facial third with the middle third of the proximal surface. There is only one exception to the rules above, which relates to the contact area between the first and second molar, whose location is in the middle third of the proximal surfaces [1].

The contact areas divide the interproximal dental space into four embrasures (gingival, occlusal, facial, and lingual) whose sizes and shapes depend on the location of the contact area. Their features are, essentially, consequences of the function of the tooth. As they move from the posterior toward the anterior, the occlusal embrasures decrease in size while the gingival ones increase [1]. Furthermore, the facial ones are wider and shallower than the oral ones. The marginal ridges of two neighboring teeth have the same height, and the proximal slopes show the same inclination and facial–oral symmetry [1,2].

The proximal contact area has dynamic characteristics influenced by tooth type, location on the arches, time of the day, and chewing [3]. Considering the above, as measurements of the passing-through force are difficult to obtain under clinical conditions, in vitro studies are preferred despite their shortcomings.

The physiological attrition process drives a decrease in the interproximal contact tightness over time, allowing unwanted complaints to arise at variable periods of time after the moment of the restorative intervention. Considering all this, it is very important to evaluate (at the end of the working session, as well as periodically during check-ups), using dental floss and radiological exams, the proximal areas and the intensity of the contact between them. 

The restoration of the proximal surfaces frequently presents different issues, with the correct choice of biomaterials, instrumentation, and working technique being essential to ensuring the quality and longevity of a restoration.

At present, various adherent biomaterials are available for direct restorations, the selection of which is performed according to the features of the clinical case and their physical/chemical properties.

There are several classifications of these biomaterials, depending on certain factors. One of the most important factors is the type of bonding to the hard dental tissues. In this context, the restorative materials may have a chemical bond (the glass ionomer cements), a micromechanical and adhesive connection (the composite resins), or a mixture of these (i.e., hybrids such as resin-modified glass ionomers, compomers, alkasites, and other materials) [4].

From the clinical point of view, the following aspects are frequently considered by operators: mechanical/wear resistance, elasticity, the volume variations after setting, esthetics, delivery presentation (syringes or capsules) with adapted injection or hand instrument application techniques, bulk or incremental layering, setting time, and so on.

Restorative glass ionomer cements (GICs) are, basically, a result of the combination of polyacrylic acid liquid and a fluor aluminosilicate glass powder. They have a medium consistency, allow for bulk application (no matter the depth of the cavity), and are self-setting, while providing a limited window for application, contouring, carving, and so on. They also need a protective coat after finishing.

Composite resins include an organic matrix, inorganic fillers, and a silane coating for bonding. The properties of the material are a consequence of the combination of characteristics of each component. Generally, the organic matrix is responsible for its fluidity, elasticity, and polymerization shrinkage, while the filler determines the wear resistance. The choice of filler results in a large range of variation among modern composite resins, and a new classification has been suggested, with ultra-low fill, low-fill, and compact resin composites [5].

Nano-fillers are considered to be one of the most important developments in this context; they allow for the incorporation of a large amount of filler and provide high wear resistance, clinically acceptable flowability, and a low quantity of organic matrix, thus significantly decreasing polymerization-related shrinkage. This enables bulk applications of a certain thickness for light-curable composite resin or close hybrids, without the need for the layering technique. Fundamentally, bulk-fill materials have a lower polymerization shrinkage but a higher translucency than incremental resins [6], and flowable bulk-fill resins require shorter light-curing times than those with a higher viscosity [7].

Hybrid biomaterials have combined properties, with resin-modified glass ionomers being closer to the glass ionomer cements and compomers/giomers being closer to composite resins. New categories, such as alkasites, have been introduced in order to leverage the benefits provided by new ingredients which might have clinical contributions.

Along with the restorative biomaterial, the conformation system (matrix/band and retainer) and type of wedge also play essential roles in ensuring that a correct restoration is achieved. Conformation systems are classified according to various criteria. The ‘’perfect’’ matrix should be thin, contoured, and colored but transparent, while also characterized by reduced deformation risk and ease of application. They may be sectional or circumferential, being made of plastic or metal [4].

The wedges are also very important, both at the beginning of the working protocol (for pre-wedging) and during the restoration stage (for correct adaptation of the matrix to the remaining hard structure) [4].

Considering all these factors, a proper contact area can be ensured through the implementation of pre-wedging (with wedges or separation rings), spatial evaluation, interproximal clearance and correct selection, and positioning and stabilization of the matrix [8]. Sometimes, in a case where the proximal loss of lateral teeth is too wide for pre-wedging, a special instrument for the contact area can be used within the material application step [9].

The clinical method for assessing the contact areas implies the use of dental floss, which should pass with an acoustic snap. This method has a high degree of subjectivity and, for this reason, in vitro studies—which are more objective—present a better trend of development within this field, despite a lack of natural conditions. Several devices have been designed in order to evaluate the intensity of the interproximal area in non-clinical environments using simulation models with plastic/ivorine teeth, offering the possibility of standardized and reproducible conditions [10,11,12].

The purpose of this in vitro study was to compare the tightness of the contact area after restoring two adjacent proximal surfaces with one of the following bulk-application biomaterials: a hybrid glass ionomer cement (GIC)—Equia Forte^TM^ HT Fil (GC, Tokyo, Japan), an alkasite—Cention^®^ Forte (IVOCLAR VIVADENT AG, Schaan, Liechtenstein), a nano-hybrid ORMOCER^®^—Admira Fusion x-tra (VOCO GmbH, Cuxhaven, Germany), and a nano-filler composite resin—3M^TM^Filtek^TM^ One Bulk Fill (St. Paul, MN, USA). The intensity of the interproximal contact was measured at the beginning and end of the working session, as well as after 7 days and 14 days.

The first null hypothesis was that each biomaterial generates contact areas with the same tightness as those observed for sound surfaces, while the second null hypothesis is that all four biomaterials provide proximal contact areas with the same intensity.

## 2. Materials and Methods

**Figure 1:**
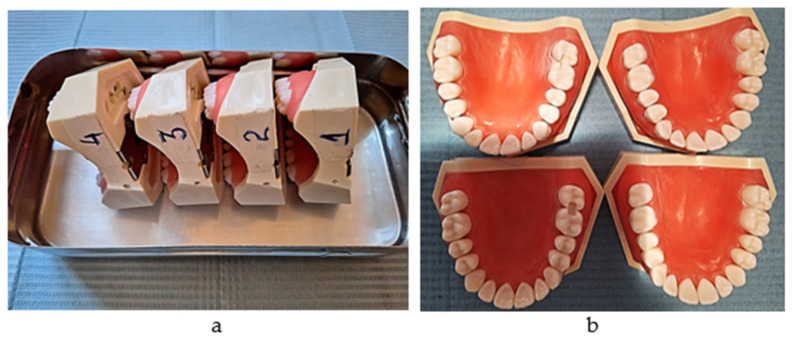
(**a**) The simulation models (serial numbers 1,2,3,4); (**b**) the proximal boxes on molars (one group out of 40).

A total of 160 plastic first molars and their adjacent posterior teeth from the maxillary right quadrant were divided equally into four soft gum simulation models; for this purpose, 8011 (Anatomika) models were used in this study. Distal boxes on the first molars and mesial boxes on the second ones were prepared (Figure 1a,b).

For every 40 pairs of molars in each simulation model, the tightness of the interproximal contact was measured between the sound proximal surfaces and then at 3 different times after preparation and restoration. For every model, the proximal boxes were restored using the same instruments but with a different restorative biomaterial.

Every time a tooth was positioned into the simulation model, a new screw was used. In order to obtain correct repositioning, for every pair of molars, an occlusal key of the sound and adjacent teeth was developed at the beginning of the working protocol.

The working protocol for each pair was completed by 4 operators under standardized conditions, each one completing 25% of every group of 40 pairs belonging to one simulation model.

All operators followed some of the conventional clinical steps, including pre-wedging; preparation of the proximal cavities; cleaning of the cut and uncut surfaces, matrix system and wedge application; use of adhesive systems; bulk application of the biomaterials and application of protection coating layer.

The steps of the protocol were as follows:

1. A torque ratchet of 10–45 Ncm^2^ (Alpha Bio, Modi’in, Israel) was used to tighten every tooth with a screwing force of 15 N.
2. Pre-wedging with maple-wooden white wedges (Fixing Wooden Wedges No.1080; TOR VM, Moscow, Russia) was applied into the oral embrasure. Pre-wedging is essential to compensate for the thickness of the metal bands to be used in the restorative stage.
3. Preparation of the cavities. The distal cavity was completed first, followed by the mesial preparation. The preparation of each cavity was carried out at medium speed with round-shaped excavation carbide burs; the finishing steps being sorted out with yellow abrasive fissure rotative instruments. The cavities had box-like aspects with 4 mm depth in all directions with rounded inner angles, with no undermined surfaces. The edges were leveled and unbeveled, with external rounded edges. An Iwanson-type tool (Medenta, Ladbergen, Germany) for evaluation of the thickness of dental crowns was used to measure the limits of the preparations.
4. Cleaning of the cut and the adjacent uncut surfaces of both cavities was carried out with physiological serum and drying was carried out gently with air and cotton pellets.
5. Treatment of the cut surfaces was carried out according to the type of restorative biomaterial to be used. A specific adhesive system was used for each resin-based material. These were applied before the matrix system and wedge.
6. Application of the coronal conformation system. In all situations, the all-in-one matrix sectional ring myClip 2.0 (Polydentia, Lugano, Switzerland) with small purple tines and LumiContrast sectional matrices (Molar; thickness—0.04 mm, height—6.4 mm) (Polydentia, Lugano, Switzerland) were used. For every pair of teeth, two matrices were placed simultaneously back-to-back and stabilized with the matrix ring and two new maple-wooden white wedges (Fixing Wooden Wedges No.1080; TOR VM, Moscow, Russia) applied in the gingival embrasure from both facial and oral directions with no overlap.
7. The material was applied via an injection technique in the mesial cavity of the second molar, with carving performed following the residual anatomy of the tooth and removing the excess.
8. The wedges and matrix ring were successively removed.
9. After complete set, the matrix for the mesial surface was removed.
10. The matrix ring and wedges were successively replaced.
11. No burnishing of the matrix for the distal surface was performed.
12. The material was applied with an injection technique in the distal cavity of the first molar, with carving performed following the residual anatomy of the tooth and removal of the excess.
13. After complete set, the wedges, matrix ring, and matrix band for the distal surface were successively removed.
14. No finishing/polishing procedures were performed.
15. Application of coat, where necessary, was carried out.

The biomaterials chosen for the study were intentionally selected as belonging to different classes of materials with indications for the restoration of large proximal cavities: a hybrid glass ionomer cement (GIC)—Equia Forte^TM^ HT Fil (GC, Tokyo, Japan), an alkasite—Cention^®^ Forte (IVOCLAR VIVADENT AG, Schaan, Liechtenstein), a nano-hybrid ORMOCER^®^—Admira Fusion x-tra (VOCO GmbH, Cuxhaven, Germany), and a nano-filler composite resin—3M^TM^Filtek^TM^ One Bulk Fill (St. Paul, MN, USA). They were all bulk-applied and inserted into the cavities using the injection technique.

During the restorative stage, every 40 pairs of adjacent cavities belonging to one of the four models was restored using one of the four biomaterials.

When light curing was needed, an LED curing light lamp (Premium Plus-BlueTech, CO2-M Mini LED, 440–480 nm, max. power of 1200 mW/cm^2^) was used.

The following protocol steps depended on the restorative biomaterial.

1. Equia Forte^TM^ HT Fil (GC, Tokyo, Japan) (Table 1) is a bulk fill glass ionomer hybrid which is suitable for direct long-term restorations. It has capsule delivery for mixers, and the working technique was followed carefully, according to the manufacturer’s instructions.

GC cocoa butter was applied on the inside of the matrices.

**Figure 2:**
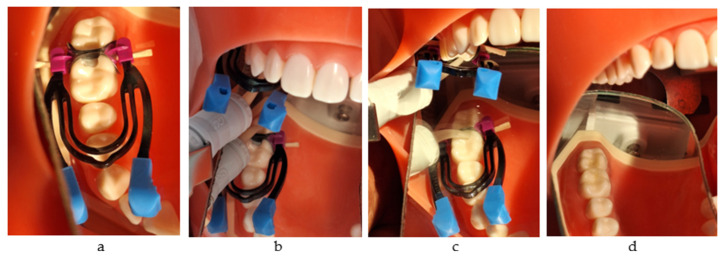
The restorative working protocol with Equia Forte HT Fil (GC): (**a**) application of the matrix system; (**b**) injection into the mesial box; (**c**) injection into the distal box; (**d**) the final restorations.

The application protocol included tapping the capsule to loosen the powder, depressing the plunger and holding down firmly for 2 s, mixing for 10 s, inserting into the applier, clicking the latter twice to prime the capsule, dispensing into the cavity with the injection technique within 10 s, packing, removing the excess, and contouring using the Optra Sculpt NG instrument (Ivoclar Vivadent AG, Schaan, Liechtenstein) with ball and chisel non-stick attachment tips. After the material had completely set, a probe was used to slightly separate the matrix from the material. The total working time was 1 min 30 s, and final finishing was completed after 2 min 30 s from the start of mixing. Then, Equia Forte Coat was applied and light-cured for 20 s (Figure 2a–d).

2. Cention^®^ Forte (Ivoclar Vivadent AG, Schaan, Liechtenstein) is an alkasite-type biomaterial, namely, a resin-based modified composite restorative with alkaline fillers which are responsible for leaching acid-neutralizing ions. We used color A2. It is a self-curing radiopaque filling material with light-curing option in the wavelength range of 400–500 nm (Table 2).

Cention^®^ Forte (Ivoclar Vivadent AG, Schaan, Liechtenstein) requires an adhesive system.

Cention^®^ Primer (Ivoclar Vivadent AG, Schaan, Liechtenstein) is a self-curing primer especially designed to be used with Cention^®^ Forte. It consists of coated single-use applicators and a bottle with liquid. One single-use applicator was dipped into a drop of primer and stirred for 5 s until the tip appeared yellow. The inner surface of the cavity was then scrubbed for 10 s and dried out with oil-free compressed air until a steady lining layer was formed.

The application protocol included activating the capsule by pressing the plunger on a flat surface, inserting into the capsule mixer and mixing for 15 s at room temperature (22–26 °C), inserting into the applier after activating it, injecting, packing, removing the excess, and carving within a total time of 2 min since the start of mixing. The condensing and contouring of the material were completed with the Optra Sculpt NR instrument (Ivoclar Vivadent AG, Schaan, Liechtenstein) with ball and chisel non-stick attachment tips. The 4 mm thick material was also light-cured for 20 s, from the occlusal direction.

**Figure 3:**
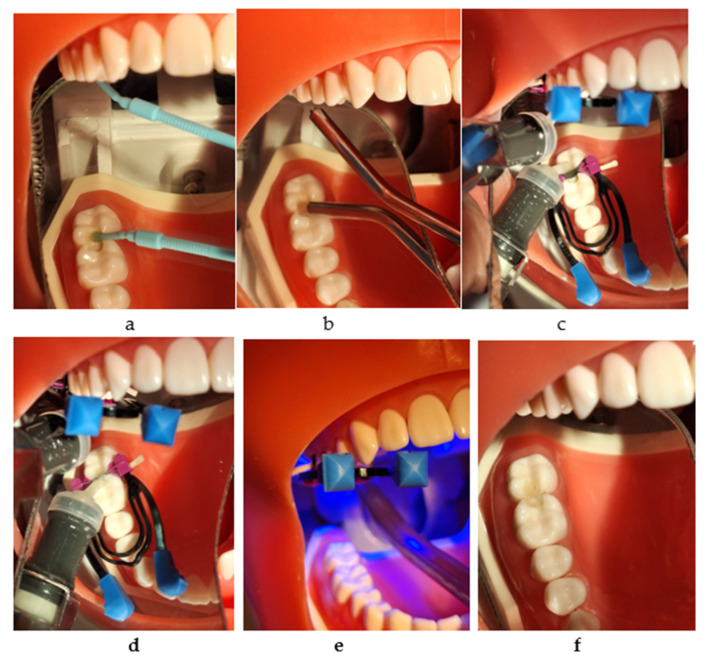
The restorative working protocol with Cention^®^ Forte (Ivoclar Vivadent): (**a**) applying Cention^®^ Primer (Ivoclar Vivadent); (**b**) drying with air; (**c**) injection into the mesial box and light curing; (**d**) injection into the distal box; (**e**) light curing for 20 s from all directions; (**f**) the final restorations.

For every proximal wall, after removing the wedge, matrix ring, and matrix, the restoration was light-cured for 20 s from the oral and buccal directions (Figure 3a–f).

3. Admira Fusion x-tra (VOCO GmbH, Cuxhaven, Germany) is a bulk ORMOCER^®^-based biomaterial (Table 3) with nano-hybrid filler (84% *w*/*w*) in a universal shade. It has methacrylate and BHT, and is delivered in syringes and caps, providing a cure depth of 4 mm. We used caps, applying the material via injection. The light-curing process takes 20 s from the occlusal direction and, after removing the metal matrix, 20 s from oral and buccal directions [15].

**Figure 4:**
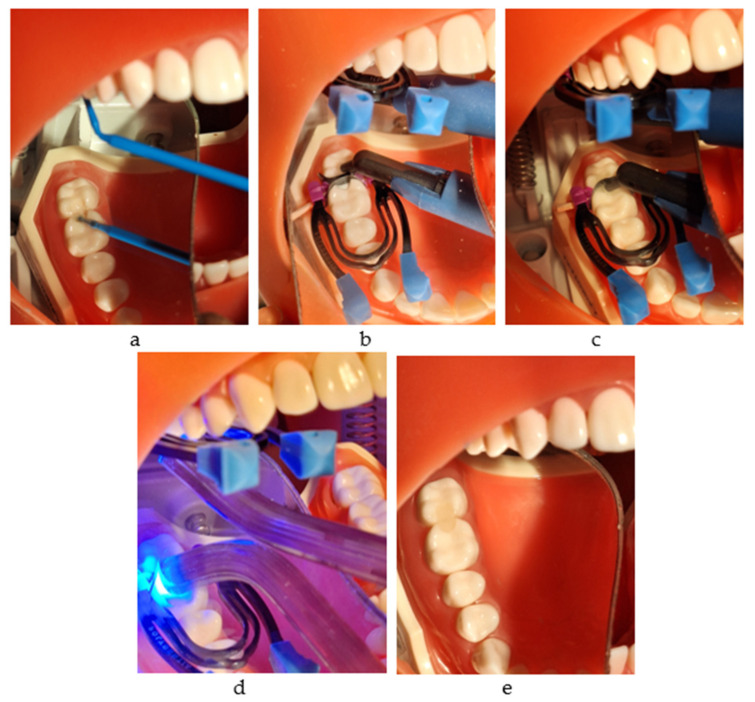
The restorative working protocol with Admira Fusion x-tra (VOCO): (**a**) applying Futurabond U and drying with air; (**b**) injection into the mesial box and light curing; (**c**) injection into the distal box; (**d**) light curing for 20 s from all directions; (**e**) the final restorations.

It may be used with any adhesive system, Futurabond U (VOCO GmbH, Cuxhaven, Germany) being the choice for this study. It is a dual-curing universal adhesive with a single-dose delivery system. In this study, we applied it with a self-etch technique. After pressing on the designated area, the applicator tip was immersed into the mixing reservoir, and one layer was applied, massaging the surface for 20 s. After drying for 5 s, the layer was light-cured for 10 s (Figure 4a–e).

4. 3M^TM^Filtek^TM^ One Bulk Fill (St. Paul, MN, USA) is a nano-filler composite resin designed for bulk application up to 5 mm. It is delivered in capsules, and for a 4 mm layer, it was necessary to light-cure for 10 s first from occlusal direction and, after removing the metal matrix, 10 s from oral and buccal directions.

**Figure 5:**
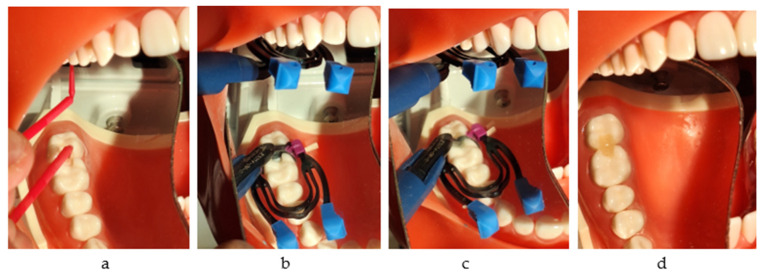
The restorative working protocol with 3M^TM^Filtek^TM^ One Bulk Fill: (**a**) applying Adper Prompt L-Pop Self-Etch Adhesive 3M and drying with air; (**b**) injection into the mesial box and light curing; (**c**) injection into the distal box; (**d**) the final restorations.

The adhesive system was the associated Adper Prompt L-Pop Self-Etch Adhesive 3M (St. Paul, MN, USA). This is a self-etch adhesive provided in L-Pop blisters for one-time use only, in a one-step application. The blister has three reservoirs (red, yellow, and green), which are squeezed in turn from the outer end in the direction of the applicator. Once the green reservoir was full, the disposable applicator was turned back and forth in the fluid for proper mixing until it became homogenously yellow. The adhesive was brushed and massaged with pressure on the surface of the cavity for 15 s. The layer was thinned by drying out with a gentle air stream. A new layer was applied without scrubbing and light-cured for 10 s (Table 4, Figure 5a–d).

**Figure 6:**
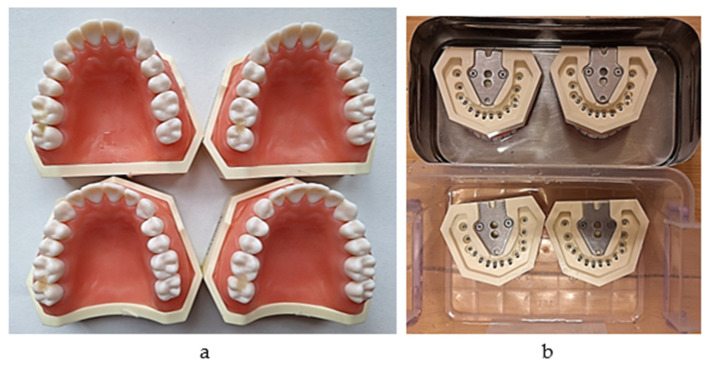
(**a**) The proximal restorations on molars (one group out of 40); (**b**) immersion in physiological serum in between measurements.

The intensity of the interproximal contact was measured at the beginning, on sound molars, for each of the 40 pairs per one model, and at 3 different times after restoration: at the end of the working session, after 7 days, and after 14 days. After the first measurement, the restored teeth were kept immersed in physiological serum until the following measurement was performed (Figure 6a,b).

The measurements were made using dental floss and a custom-made system that we designed and used in a previous study [10]. Compared to other types of devices [11,12], we believe that this measurement system provides a different approach in order to objectively evaluate the tightness of proximal contact.

**Figure 7:**
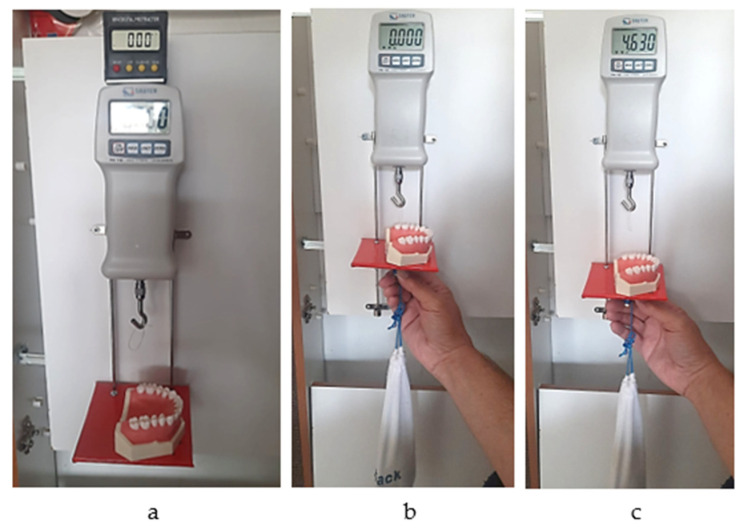
(**a**) Checking the vertical position; (**b**) initial moment of the measurement; (**c**) final moment of the measurement (the peak value of the passing-through force).

The system consists of a 10 N dynamometer (Sauter FK 10; Kern, Germany) attached to a melamine sheet fixed on a wall, a mobile horizontal pad for the simulation model to be fixed on that can slide gravitationally on two metallic vertical rails, and a mass of 850 g which provides constant downward vertical traction force. A mini digital protractor inclinometer electronic level is used to check the positions of the dynamometer and the pad every time a measurement is made. A new 3.5 cm-long loop of dental floss (Sensodyne Expanding Floss) was used for each measurement. The model was fixed with a screw on the horizontal plate in the same position (previously marked), such that each measurement was performed under the same conditions (Figure 7a–c).

The peak values of the forces required to pull the dental floss through the interdental (sound and restored) areas were recorded. For each of the 4 biomaterials, 3 measurements (every 5 min) were made for each of the 40 (sound and restored) contact areas at the end of the procedure, after 7 days, and after 14 days.

As the working protocol had been carried out by 4 practitioners, the measurements were performed by 4 other operators who were not aware of the types of biomaterials that had been used.

A custom program composed in Excel (MS Office Professional Plus 2021 for Windows) was used in order to collect data. The sample size was represented by 40 teeth for each set of materials.

Statistical analysis was performed with the “R” statistical computing program (version 4.2.3-Shortstop Beagle) using one-way analysis of variance (ANOVA), followed by Tukey’s multiple comparisons of means test to determine the differences in proximal contact tightness between groups. Each group of forty teeth passed through three sets of measurements. The data samples were scrutinized using the Shapiro–Wilk normality test and the corresponding residual histograms, due to the large number of measurements. The level of significance was set at *p* < 0.05. The power calculation performed for ANOVA after restoration showed a large difference effect, according to Cohen’s general guide.

## 3. Results

**Figure 8:**
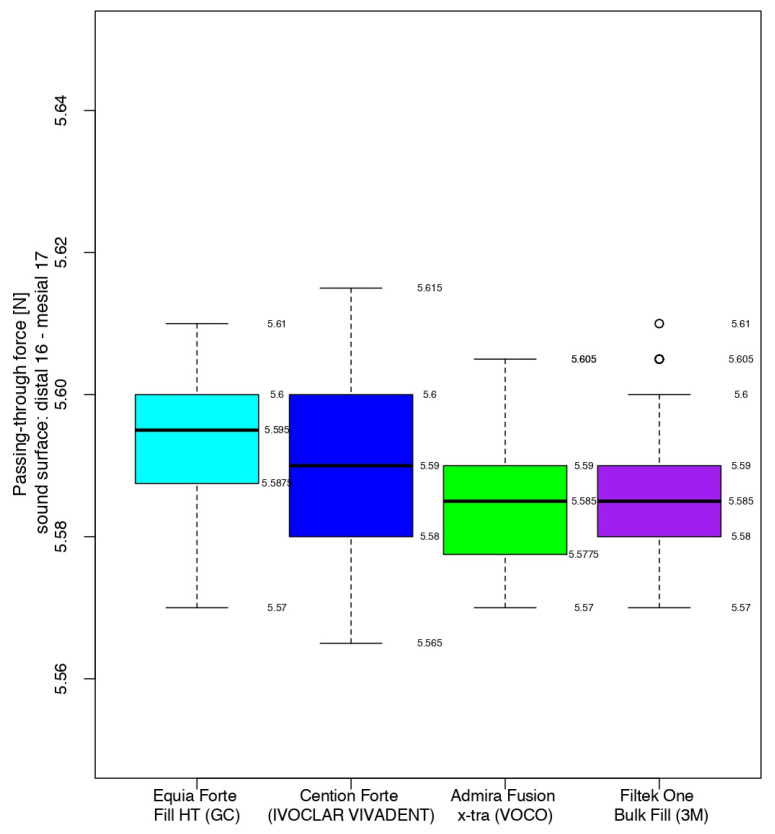
The intensity of the contact areas of the sound molars.

The tightness of the proximal contact was first measured between the first and second sound right maxillary molars. Close values of the passing-through forces and standard deviations were recorded between all teeth of the four models (Figure 8).

Compared to the sound proximal surfaces, the measurements of the passing-through forces for the restored contact areas using Equia Forte^TM^ HT Fil showed a significantly increased mean value (4.07 N higher) at the end of the working protocol. After 7 days, the mean value was 4.09 N higher. After 14 days, the passing-through force difference was 4.11 N higher (Table 5). Compared to the sound surfaces, the number of standard deviations at the three measurement times were 408.55, 426.82, and 423.73, respectively.

At the end of the working protocol, 7 days after, and 14 days after the restorative procedure with Cention^®^ Forte, the mean intensity of the contact areas was 3.30 N, 3.50 N, and 3.56 N higher, respectively, when compared to the sound proximal surfaces (Table 6). Compared to the sound surfaces, the number of standard deviations at the three measurement times were 219.09, 259.02, and 257.08, respectively.

The passing-through forces of the restored contact areas using Admira Fusion x-tra showed a mean value that was increased by 4.11 N at the end of the working protocol when compared to the sound proximal surfaces. After 7 days, the mean value was 0.02 N higher, and after 14 days, the passing-through force difference was also 4.11 N higher (Table 7). Compared to the sound surfaces, the number of standard deviations at the three measurement times were 408.55, 426.82 and 423.73, respectively.

Immediately after and 7 days after restoration with 3M^TM^Filtek^TM^ One Bulk Fill, the mean intensity of the contact areas was 4.08 N and 4.09 N, respectively, higher than that of the sound ones. After 14 days, a decrease of 0.02 was recorded with respect to the same value 7 days before (Table 8). Compared to the sound surfaces, the number of standard deviations at the three measurement times were 354.21, 396.41, and 322.54, respectively.

At the end of the working protocol, the closest values between the four materials were recorded for the restorations completed with the hybrid glass ionomer cement and the nano-filled composite resin. Namely, 50% of the passing-through force values ranged from 9.660 to 9.670 and from 9.665 to 9.675 for Equia Forte^TM^ HT Fil and 3M^TM^Filtek^TM^ One Bulk Fill, respectively, the median value being only 0.005 higher for the latter.

The highest values (9.685–9.695) were registered for half of the measurements in the contact areas restored with Admira Fusion x-tra, the median value having higher values than the glass ionomer cement and the composite resin, by 0.030 and 0.025, respectively.

The median value for Cention^®^ Forte was significantly lower, with half of the values being in the interval 8.885–8.900, while the higher quartile (25%) presented data between 8.900 and 8.905.

**Figure 9:**
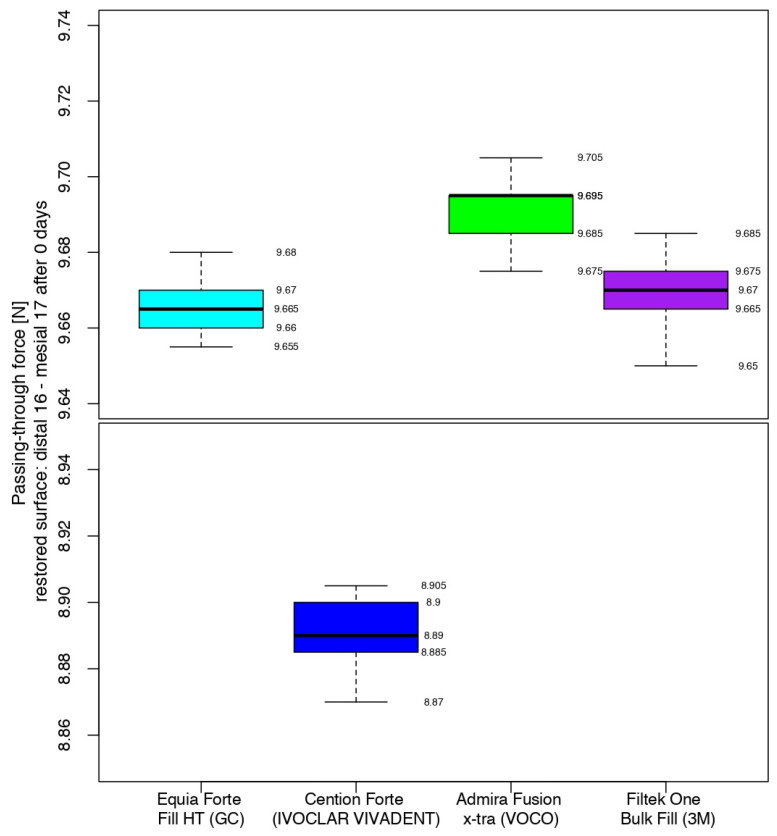
Comparison of the intensity of the contact areas at the end of the working protocol.

Therefore, immediately after restoration, the alkasite recorded the closest values (median value of 8.890) to those of the sound contact areas (median value of 5.590), but with a significant difference with respect to the other three biomaterials. Analyzing their box-and-whisker plots, 50% of all passing-through force values varied between 9.660 and 9.695 versus 5.577 and 5.600 for the sound surfaces (Figure 9).

**Figure 10:**
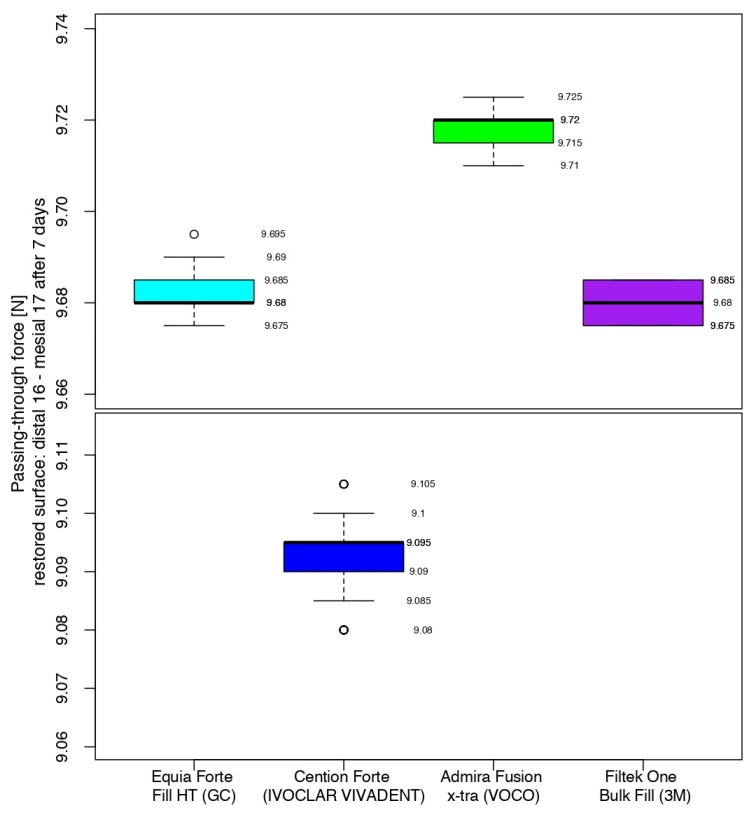
Comparison of the intensity of the contact areas after 7 days.

After 7 days, the comparison between the composites showed that the situation was similar for the glass ionomer cement and the nano-filled composite resin, with the middle quartiles (50%) ranging from 9.680 to 9.685 N (Equia Forte^TM^ HT Fil) and from 9.675 to 9.685 N (3M^TM^Filtek^TM^ One Bulk Fill). For Admira Fusion x-tra (Voco), half of the values were in the range of 9.715–9.720 N, with a median value 0.025 higher than its position in the box plot immediately after the restorative process. Cention^®^ Forte recorded a median value 0.105 higher than that at the end of the working procedure, with the highest value of the middle quartile being 0.580 and 0.615 under the lowest values of the glass ionomer cement/nano-filled composite resin and the Ormocer, respectively (Figure 10).

**Figure 11:**
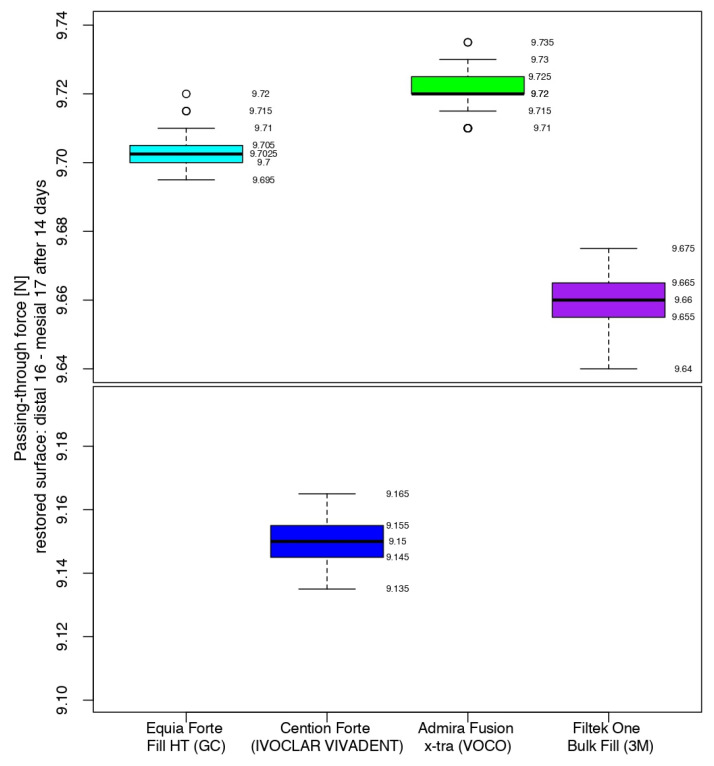
Comparison of the intensity of the contact areas after 14 days.

After 14 days, the measurements of the restored contact areas with 3M^TM^Filtek^TM^ One Bulk Fill showed close values to those obtained in the previous measurements (middle quartile ranging from 9.655 to 9.665 N), with the first median value being lower than the previous one. The values recorded for Admira Fusion x-tra had the same median value as 7 days before, with 50% of the values between 9.720 N and 9.725 N. The contact areas restored using Equia Forte^TM^ HT Fil had a 0.07 higher difference for the median than in the previous measurements. Cention^®^ Forte-restored contact areas recorded the second-highest difference in value for the median between measurements, when considering restored surfaces using the same biomaterial (Figure 11).

**Figure 12:**
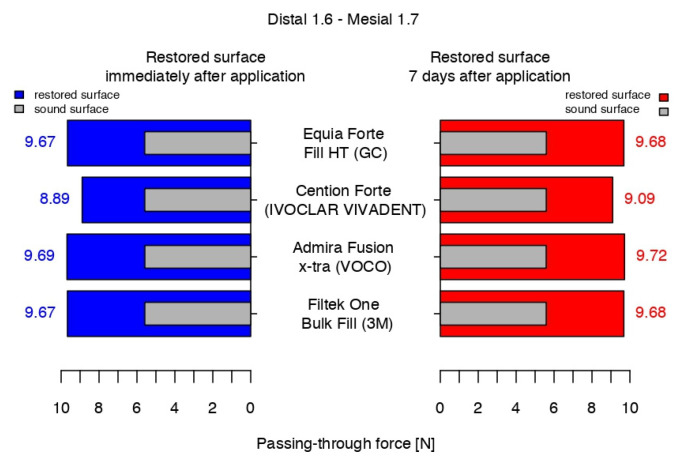
The passing-through forces values immediately after application and at 7 days after application versus before preparation.

At the end of the working protocol, the results indicated that the passing-through force values for all restored contact areas were significantly higher than those of the sound proximal surfaces and, in the comparison between them, all biomaterials recorded close values, except for the Cention^®^ Forte (Figure 12).

After 7 days, the intensity of the contact areas restored using Equia Forte^TM^ HT Fil and 3M^TM^Filtek^TM^ One Bulk Fill registered similar statistical data, with a very slight difference versus the previous measurement. Admira Fusion x-tra had the highest value but was still close to the latter. The values for Cention^®^ Forte were significantly lower, compared to the other biomaterials.

**Figure 13:**
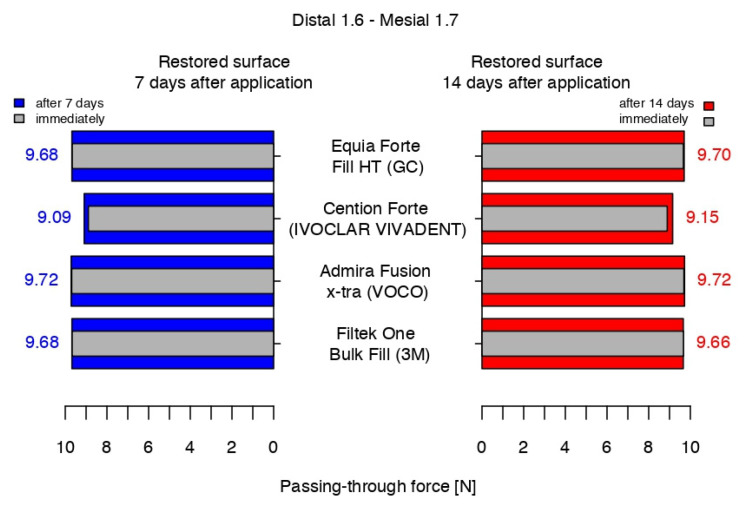
The passing-through force values at 7 and 14 days after versus immediately after the working protocol.

After 14 days, compared to 7 days before, the median value of Admira Fusion x-tra was stable, while the values for Equia Forte^TM^ HT Fil and 3M^TM^Filtek^TM^ One Bulk Fill indicated a slight increase of 0.2 N and a decrease of 0.2 N, respectively. With a median value only 0.06 higher than that measured a week before, a significant difference was registered again between the median value of Cention^®^ Forte and the average data of the others (Figure 13).

**Figure 14:**
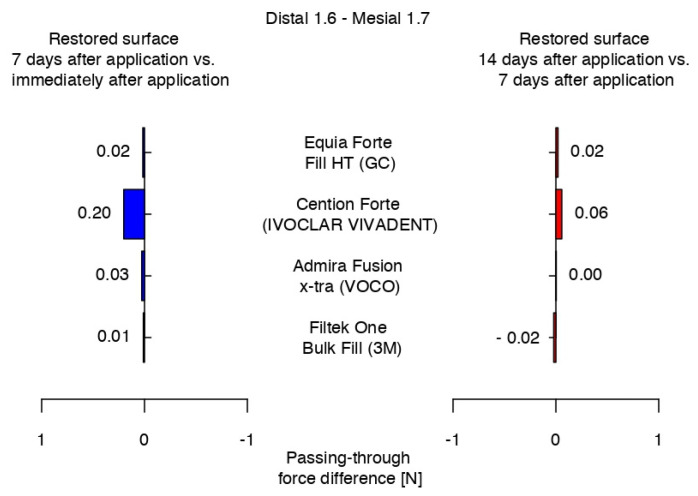
Variations in the intensity of the contact areas between measurements.

The highest difference in the passing-through force was recorded for Cention^®^ Forte between the measurements after 7 days and immediately after application, and the only negative value was registered for 3M^TM^Filtek^TM^ One Bulk Fill between the values recorded at 7 and 14 days after the restorative protocol. Equia Forte^TM^ HT Fil presented a constant difference in the passing-through force values between measurements of the restored surfaces, while Admira Fusion x-tra showed very close range of values in the middle quartile at 7 and 14 days, presenting the same median (Figure 14).

## 4. Discussion

Direct restoration of two adjacent proximal walls with large loss of hard tissue in the molar region is generally a difficult and time-consuming task, even for experienced practitioners. For this reason, a clever choice of the technique, instrumentation, and biomaterials to be used is essential.

As mentioned above, the high degree of subjectivity regarding clinical assessment of the contact area with the use of dental floss explains why, in this field of interest, in vitro studies are more numerous, as they offer standardized and reproductible conditions [12,17].

However, despite their initial objectivity, they naturally have several shortcomings related to the use of artificial teeth models in a non-clinical environment. It is understandable that these aspects may influence the adhesion, polymerization shrinkage, and mechanical properties of the materials. Furthermore, the short duration of the studies, along with the lack of humidity, masticatory loads, and variations in temperature hinder the ability to obtain realistic information about their evolution over time. Despite these shortcomings, the numerous studies within this field of interest have provided results which are considered to have potential clinical significance.

Their general aims have been the study of biomaterials and auxiliary instruments used within the working protocol, focusing on the evaluation of separation devices and matrix systems.

Several studies have compared the efficiency of different types of matrices through measuring the tightness of the interproximal contact after restoration, their results having significant clinical value and providing useful information for practitioners. For example, a significant number of in vitro studies have shown that the use of sectional matrices combined with separation rings generated tighter proximal contacts, compared to circumferential bands or conventional matrix systems [10,12], and the use of separation rings with sectional matrices helped in providing superior contacts [17,18,19,20].

According to a clinical study performed by Bogovska-Gigova and Hristov, a proper proximal contour of the restorations on primary teeth is influenced more by the type of the matrix than by the filling material or application technique [21].

In this context, our intention was to use one type of conformation system/wedge and focus our study on comparing the efficiency of four different categories of materials for restoration in a frequent and complicated clinical situation, namely, large, face-to-face hard tissue losses on molars. The material was selected according to the manufacturer’s indications, having specific properties that recommended/indicated them for direct restorations of large proximal loss of dental tissue in the posterior region.

The choice of materials (a glass ionomer cement, an alkasite, an ORMOCER-based resin, a nano-filler resin) was also informed by the fact that we found a very limited number of studies that have solely focused on comparison between categories of materials, most of them being focused on comparing the efficiency of different types of composite resins. This aspect is understandable considering that they are frequently the first choice for most practitioners. Still, polymerization shrinkage remains a problem that may influence—considering the subject of our study—the intensity of the proximal contact. Low polymerization shrinkage and low-intensity curing units have been generally recommended to ensure good tightness of the contact area [22].

Polymerization shrinkage has been considered the major problem when it comes to methacrylate-based composite resins, its cause being the shortening of the distances between monomeric units in the polymer compared to intermolecular distances in uncured monomers [7]. For the same generation and type of composite resin, flowable materials have been shown to possess higher values in terms of shrinkage strain and stress [23].

Clinically, a reduction in polymerization shrinkage can be achieved through incremental application, avoiding the connection of the opposite walls through one layer. If performing bulk application, the resins should have a certain percentage of nano-filler. Still, according to Oliveira et al., the bulk-filling technique for resin-based biomaterials generated similar proximal contact intensity when compared to that associated with an incremental filling [24].

All four biomaterials used in this study have improved chemical and physical qualities, according to their manufacturers.

Despite their completely different compositions, they all had in common the bulk-application mode, and they were all applied using an injection technique. It is already well known that the consistency of a material influences its handling characteristics, with fluid materials being better adapted to the cavity walls, while paste materials are handled easier when using modelling liquids [25]. Considering the effect of viscosity on the quality of proximal tightness, several conclusions have been drawn. According to El-Shamy et al., bulk composites with high viscosity and composites that use sonic energy to lower their consistency during placement generated restorations with tighter contact areas than flowable ones [26]. The results of other clinical studies have also demonstrated that restorations with different types of flowable resins presented decreased proximal contact strength over time [27,28,29].

Considering the importance of this aspect, we aimed to gather biomaterials belonging to completely different categories but with a rather close and higher consistency, as well as allowing the injection technique to be used with no need for modelling agents. The consistency of our materials varied from creamy to injectable paste in the following order: glass ionomer cement, alkasite, ORMOCER^®^-based resin, and nano-filled resin.

Equia Forte ^TM^ HT Fil (GC, Tokyo, Japan) is a long-term bulk-fill glass hybrid restorative system with a capsule delivery system for mixers. As a glass ionomer cement, the essential aspect lies in the chemical connection with the hard tissues, thus providing very good marginal sealing. This product, which is already well-known on the dental market, contains ultra-fine highly reactive glass and high-molecular-weight polyacrylic acid powders, providing improved fluidity, non-sticky handling, and enhanced mechanical properties which make it suitable for stress-bearing areas. It is also better-looking, due to the adapted refractive index of the fillers with respect to that of the matrix. As a result, it has high flexural strength (45.1 MPa), compressive strength (207.58 MPa), and modulus of elasticity (16.8 GPa), as well as high translucency (55.9). The bulk application and capsular delivery ensure a fast injection procedure, leading to an important reduction in the occurrence of technical errors [13].

Cention^®^ Forte (IVOCLAR VIVADENT AG, Schaan, Liechtenstein) is a hybrid material which aims to gather many benefits into one product. It is a combination of glass ionomer cement, composite resin, and calcium hydroxide-releasing product, leveraging the advantages of each. It releases fluoride, calcium, and hydroxide ions, with its ion release performance being much higher at an acidic pH value than at a neutral value. As a consequence, acid attacks are neutralized more quickly, promoting efficient remineralization and preventing demineralization. It also has a flexural strength higher than 100 MPa, making it suitable for long-lasting restorations. The material is tooth-colored, with a translucency of 11%. It has a bulk-fill fast injection application in 4 mm, with capsule delivery system for mixers. It is self-set, with a self-curing option in the wavelength range of 400–500 nm [14].

Admira Fusion x-tra (VOCO GmbH, Cuxhaven, Germany) is a bulk ORMOCER^®^-based biomaterial (Pure Silicate Technology) with a nano-hybrid filler (spherical, 20–40 nm, particles—60%, micro-/macro-particles—40%). Notably, it is free of BPA and classic monomers. It has a cure depth of 4 mm, a low initial polymerization shrinkage of 1.25% (*v*/*v*), good wear resistance, and persistent high gloss. It has only one shade with chameleonic properties due to the shape and size of the nano-fillers, which are passed through by light with no refraction or diffraction hitting the tooth structure and returning to the human eye [15].

3M^TM^Filtek^TM^ One Bulk Fill (USA) is a well-known light-curable bulk nano-filled composite resin, provided in capsules, with one-step application up to 5 mm (for LED lights with an output of 1000–2000 mW/cm^2^). It has low polymerization shrinkage due to the use of two modified monomers (AFM, AUDMA), as well as good wear resistance and persistent glossy appearance of the surface due to the nanofillers being fused into clusters. It also displays increased opacity and a proper viscosity for good marginal adaptation [16].

The working protocol for adjacent proximal large caries on posterior teeth generally requires a rather long time and is associated with a high degree of difficulty, with various available working techniques. We considered that simultaneously applying both matrices at the beginning and then removing them one by one afterwards is the most appropriate technical method. The custom-made system for measurement was custom-designed by us for use in a previous study, the conclusions of which agreed with other results obtained in this field of interest. Although its use requires more time and increased attention from the operator, it is essential to underline that it was designed in an attempt to closely emulate—as much as possible—the natural clinical use of dental floss [10].

At the end of the working protocol, the passing-through force of all restored contact areas showed significantly higher values than those of the sound proximal surfaces, with all biomaterials presenting highly similar values, except for the Cention^®^ Forte.

After 7 days, the intensity of the contact areas restored using Equia Forte^TM^ HT Fil and 3M^TM^Filtek^TM^ One Bulk Fill registered similar values to those recorded at the end of the treatment session, while Admira Fusion x-tra presented the highest value (close to that of the previous measurement) and Cention^®^ Forte obtained significantly lower values compared to the others.

After 14 days, compared to a week before, the results indicated a similar trend.

From certain aspects, our results have elements in common with those of the other (although limited) studies in this field of interest.

The conclusions of systematic reviews focused on the evaluation of methods for obtaining increased proximal tightness indicated the good association of sectional matrices (with separation rings) and composite resin with higher viscosity [30]. The use of sectional matrices (with separation rings), rather than circumferential matrices, along with low polymerization shrinkage biomaterials also yielded good results [31].

Certain differences were also recorded, but it is important to underline that the biomaterials compared within this and other studies were not the same. According to Deepak and Nivedhitha, no statistically significant difference was found between Cention N (Ivoclar Vivadent) and Charisma (Kulzer) [32], while Cerdan et al. found that high-viscosity composite resins (no matter the type of application) recorded tighter contact areas than a high-viscosity glass ionomer cement [33]. A survey among practitioners even indicated that most of the participating specialists considered amalgam restorations to have better proximal contacts than resin-based ones [34].

Our results indicated that the passing-through force varied significantly for all materials when compared to the sound surfaces, while, in the comparison in between them, the highest value was obtained with the ORMOCER-based material, followed closely by the hybrid glass ionomer cement and the nano-filled resin. Although the alkasite occupied the last place, with significantly lower values, it retains an important role in pediatric dentistry [35,36,37].

Therefore, our first null hypothesis was rejected, as all biomaterials provided contact areas with different values of tightness compared those of the sound surfaces. The second one was also rejected, as the four materials generated variable tightness of the contact areas.

As clinical conditions do not allow for objective measurements within this field of interest, our study and several other in vitro studies have used artificial teeth [12,17,20,22,26], our results being recorded without consideration of variations in temperature or occlusal forces, at three relatively close moments in time. It is obvious, then, that there are certain limitations due to the lack of a natural environment and short period of the study, the subject remaining open to further investigations including natural teeth, thermal and mechanical aging, and extension of the duration of the study, potentially enabling better prediction of the longevity of the considered restorations.

Despite these shortcomings, the results of our study indicate close ranges of values for each material between the three measurement times. The numerous measurements we obtained indicated a certain pattern for each biomaterial, evidencing their restorative behaviors and allowing us to believe in the clinical relevance of the study.

## 5. Conclusions

At the end of the working protocol, the results indicated that the measurements of the intensity of the interproximal contact of all restored areas showed significant higher values than those of the sound surfaces while, in the comparison between them, Equia Forte^TM^ HT Fil (GC, Tokyo, Japan), Admira Fusion x-tra (VOCO GmbH, Cuxhaven, Germany), and 3M^TM^Filtek^TM^ One Bulk Fill (St. Paul, MN, USA) recorded close values, leaving Cention^®^ Forte (IVOCLAR VIVADENT AG, Schaan, Liechtenstein) some distance behind.

Therefore, the results may have clinical significance in terms of understanding how to choose, from the beginning of the working protocol, the most appropriate restorative material for large proximal caries on adjacent surfaces of lateral teeth.

The statistical analysis of the results validated the design of the working protocol as a reliable and reproducible measurement method. Nevertheless, further investigations with direct correlations to the mechanical and chemical factors involved in the biodynamics of the direct restorations are necessary.

The principles of the proposed method could serve as the basis for a future in vivo device to evaluate the tightness of the proximal contact areas, both at the end of the working protocol and at various following time points, providing information about the quality of the configuration of the restored proximal areas when using different techniques, instruments, and types of biomaterials.

## Figures and Tables

**Table 1:** The composition of the hybrid glass ionomer and coat [13].

Material	Ingredients	Lot Shade
Equia Forte^TM^ HT Fil (GC)	Surface-treated FAS (fluoroaluminosilicate) glass Highly reactive surface-treated fine FAS glass High-molecular-weight polyacrylic acid Polyacrylic acid Pigment	240521A A3
Equia Forte Coat (GC)	Multifunctional monomer Methacrylate Silicon dioxide Initiator MDP Stabilizer	230928A

**Table 2:** The composition of the alkasite material and adhesive system [14].

Material	Components	Lot Shade
**Cention^®^ Forte ** **(Ivoclar Vivadent)** **Cention^®^ Primer** **(Ivoclar Vivadent)**	Ca-fluorosilicate glass Ba-Al silicate glass Copolymer Ca-Ba-Al fluorosilicate glass UDMA Ytterbium trifluoride Aromatic aliphatic UDMA, DCP, and PEG-400-DMA Inorganic filler: 58–59 vol% Particle size of inorganic fillers: 0.1–7 µm HEMA MDP Bis-GMA D3MA Ethanol Methacrylate-modified polyacrylic acid Silicon dioxide Potassium hydroxide Camphorquinone	ZL12C4 A2 Z06Y4D

**Table 3:** The composition of the ORMOCER^®^-based biomaterial and adhesive system [15].

Material	Components	Lot Shade
Admira Fusion x-tra (Voco) Futurabond U (Voco)	Barium aluminum borosilicate glass ORMOCER^®^ resin Silicon dioxide Initiators Stabilizers Pigments HMA Bis-GMA HEDMA 10-MDP Urethane dimethacrylate Catalyst Silica nanoparticles Ethanol Water	2218450 U 2213706

**Table 4:** The composition of the nano-filled composite resin and adhesive system [16].

Material	Component	Lot Shade
3M™ Filtek™ One Bulk Fill Restorative Adper Prompt L-Pop Self-Etch Adhesive 3M	Silane-treated Ceramic Aromatic urethane dimethacrylate Diurethane dimethacrylate (UDMA) Silane-treated silica Ytterbium fluoride (YbF3) Water Silane-treated zirconia 1,12-Dodecane dimethycrylate (DDDMA) Ethyl 4-dimethyl aminobenzoate (EDMAB) Water 2- Hydroxyethyl methacrylate 2-propenoic acid, polymer with methylenebutanedioic acid	NE55683 A3 8798205

**Table 5:** Evaluation of the proximal contact area when using glass ionomer cement.

Equia Forte HT Fil (GC)	Sound Surface Mean ± SD	Restored Surface After Application, Mean ± SD	Restored Surface 7 Days After Application, Mean ± SD	Restored Surface 14 Days After Application, Mean ± SD	Mean Difference After Application	Mean Difference 7 Days After Application	Mean Difference 14 Days After Application	*p* Value
Passing-through force (N)	5.593 ± 0.008	9.666 ± 0.006	9.682 ± 0.005	9.703 ± 0.005	4.073 ± 0.010	4.089 ± 0.010	4.110 ± 0.010	<0.05

**Table 6:** Evaluation of the proximal contact area when using the alkasite resin.

Cention Forte (IVOCLAR VIVADENT)	Sound Surface Mean ± SD	Restored Surface After Application, Mean ± SD	Restored Surface 7 Days After Application, Mean ± SD	Restored Surface 14 Days After Application, Mean ± SD	Mean Difference After Application	Mean Difference 7 Days After Application	Mean Difference 14 Days After Application	*p* Value
Passing-through force (N)	5.589 ± 0.012	8.891 ± 0.009	9.093 ± 0.006	9.150 ± 0.007	3.302 ± 0.015	3.504 ± 0.014	3.561 ± 0.014	<0.05

**Table 7:** Evaluation of the proximal contact area when using the Ormocer-based resin.

Admira Fusion x-tra (VOCO)	Sound Surface Mean ± SD	Restored Surface After Application, Mean ± SD	Restored Surface 7 Days After Application, Mean ± SD	Restored Surface 14 Days After Application, Mean ± SD	Mean Difference After Application	Mean Difference 7 Days After Application	Mean Difference 14 Days After Application	*p* Value
Passing-through force (N)	5.585 ± 0.009	9.691 ± 0.008	9.718 ± 0.005	9.722 ± 0.005	4.107 ± 0.012	4.134 ± 0.010	4.137 ± 0.010	<0.05

**Table 8:** Evaluation of the proximal contact area when using the nano-filled composite resin.

3M Filtek One Bulk Fill	Sound Surface Mean ± SD	Restored Surface After Application, Mean ± SD	Restored Surface 7 Days After Application, Mean ± SD	Restored Surface 14 Days After Application, Mean ± SD	Mean Difference After Application	Mean Difference 7 Days After Application	Mean Difference 14 Days After Application	*p* Value
Passing-through force (N)	5.587 ± 0.009	9.670 ± 0.007	9.681 ± 0.004	9.660 ± 0.008	4.083 ± 0.012	4.094 ± 0.010	4.073 ± 0.013	<0.05

## Data Availability

The raw data supporting the conclusions of this article will be made available by the authors on request.
